# Bioengineered Living Bone Grafts—A Concise Review on Bioreactors and Production Techniques In Vitro

**DOI:** 10.3390/ijms23031765

**Published:** 2022-02-03

**Authors:** Paulina Kazimierczak, Agata Przekora

**Affiliations:** Independent Unit of Tissue Engineering and Regenerative Medicine, Medical University of Lublin, Chodzki 1, 20-093 Lublin, Poland; agata.przekora@umlub.pl

**Keywords:** mesenchymal stem cells, osteoblasts, osteogenic differentiation, three-dimensional culture, scaffold, bioreactor, bioprinting, bioink, pulsed electromagnetic fields, bone tissue engineering

## Abstract

It has been observed that bone fractures carry a risk of high mortality and morbidity. The deployment of a proper bone healing method is essential to achieve the desired success. Over the years, bone tissue engineering (BTE) has appeared to be a very promising approach aimed at restoring bone defects. The main role of the BTE is to apply new, efficient, and functional bone regeneration therapy via a combination of bone scaffolds with cells and/or healing promotive factors (e.g., growth factors and bioactive agents). The modern approach involves also the production of living bone grafts in vitro by long-term culture of cell-seeded biomaterials, often with the use of bioreactors. This review presents the most recent findings concerning biomaterials, cells, and techniques used for the production of living bone grafts under in vitro conditions. Particular attention has been given to features of known bioreactor systems currently used in BTE: perfusion bioreactors, rotating bioreactors, and spinner flask bioreactors. Although bioreactor systems are still characterized by some limitations, they are excellent platforms to form bioengineered living bone grafts in vitro for bone fracture regeneration. Moreover, the review article also describes the types of biomaterials and sources of cells that can be used in BTE as well as the role of three-dimensional bioprinting and pulsed electromagnetic fields in both bone healing and BTE.

## 1. Introduction

Mechanical support of the body, protection of internal organs, hematopoiesis, and the reservoir of ions and growth factors are the main functions of bone tissue [1]. Bone is a rigid tissue that consists of minerals (approximately 65%) and organic parts (approximately 20–25%), while the remaining portion is water (10–20%) whose amount depends on bone health, age, etc. The mineral part is composed mostly of calcium phosphate, called hydroxyapatite (85%), as well as calcium carbonate and calcium fluorite, whereas organic parts of the bone are primarily formed by type I collagen (approx. 90%) but also non-collagenous proteins (osteonectin (OCN), osteopontin (OPN), osteocalcin (OC), and bone sialoproteins (BSP)), proteoglycans, lipids, and other macromolecules [1,2]. Mechanical properties of the bone (tensile strength, Young’s modulus, and compressive strength) are determined by both mineral and organic phases [1,3]. 

Bone tissue possesses an incredible ability to regenerate and repair itself without scar formation [2]. Nevertheless, there are various bone diseases causing bone weakness and brittleness, e.g., (1) osteogenesis imperfecta, a metabolic bone disorder causing bone fragility due to defects in type 1 collagen (COL I) [4]; (2) osteoporosis, a metabolic skeletal disease characterized by microarchitectural deterioration and decreased bone mass due to hormonal deficiencies, resulting in excessive bone resorption [5]; (3) osteomalacia, which is bone disease characterized by a loss of bone mineral caused by nutritional deficiencies [6]; (4) osteomyelitis, which is an inflammatory state of bone caused by microorganism [7]; and (5) primary and metastatic cancers [1]. A decrease in bone density caused by diseases may result in partial or total loss of bone continuity caused by low-energy trauma, which is called a fragility fracture [1]. Nowadays, a great number of elderly with confirmed osteoporosis (approximately 200,000,000 patients each year) and osteoporotic fractures (approximately 9,000,000) worldwide has been observed [8]. The life expectancy of the people has risen significantly in developed countries, causing an increased incidence rate for osteoporotic fractures and thereby an increase in socioeconomic costs [2]. Bone fracture healing is a complex and multistage process which consists of four steps. The first stage is the inflammation that includes hematoma formation, release of bone morphogenetic proteins (BMPs), tumor necrosis factor-alpha (TNF-α), tumor-derived growth factor-beta (TGF-β), the platelet-derived growth factor (PDGF), and interleukins (IL-1, IL-6, IL-11, and IL-23). It also involves recruitment and migration of mesenchymal stem cells (MSCs) [9,10]. The second phase of bone healing is associated with cartilage formation and periosteal response. During this phase, neo-angiogenesis, chondrogenesis, and endochondral ossifications are observed. The third phase involves cartilage resorption and primary bone formation. Then, during the fourth phase, bone remodeling related to osteoclast activity is observed [10].

In the case of large bone loss, bone grafts are needed to support and accelerate bone fracture regeneration. It is worth noting that next to the blood, bone is the most frequently transplanted tissue [2]. There are three types of natural bone grafts: autografts, allografts, and xenografts. Autografts are considered the gold standard because of their high histocompatibility. The autografts are derived from the host bone (e.g., iliac crest and rib) and implanted into the bone-loss site. Unfortunately, restricted donor source, donor-site morbidity, infection, and pain are among the limitations of using autografts [2,11]. In turn, allografts are derived from donors being of the same species (from cadavers or living donors). Clinically, fresh-frozen bone and freeze-dried/demineralized bone are the most frequently used [12]. The allografts carry a risk of immune reaction, the possibility of infection, and possibility of disease transmission [2,11,13]. Nevertheless, it is worth noting that tissue banks perform tissue sterilization by using specific disinfection methods during the preparation and processing of allograft tissues, thereby minimalizing viral or bacterial disease transmission [14]. Moreover, a number of commercial bone allografts are available, for example, Osteocel^®^ Plus, Osteocel^®^ PRO (Nuvasive, San Diego, CA, USA), Via^®^ (Vivex Biologics Inc., Miami, FL, USA), ViviGen^®^Formable (DePuy Synthes, Raynham, MA, USA), Map3^®^ (RTI Surgical, Alachua, FL, USA), and CeLLogix (Omnia Medical, Morgantown, WV, USA) [15]. Grafts obtained from animals are called xenografts that can cause immune reactions and carry a rare risk of transmission of zoonotic diseases [2]. Among commercially available bone xenografts, the following products may be distinguished: SmartBone^®^ (IBI, Mezzo-Vico Vira, Switzerland) and i-FACTOR^®^ P-15 (Cerapedics, Westminster, CO, USA) [15]. Another approach to bone-fracture regeneration is the application of metal implants made of stainless steel or titanium alloys, such as screws, plates, and joint prostheses. The main drawbacks of this approach are high rigidity, non-degradability, and often poor osseointegration with the host tissue [13]. 

Bone fractures, such as osteoporotic fractures, and implant-associated infection are characterized by high mortality and morbidity [16,17]. Therefore, the selection of an appropriate healing method is crucial for good clinical outcomes. Thus, over the years, a growing interest in bone tissue engineering (BTE), which is a very promising approach to bone-fracture regeneration, has been observed. The aim of this paper is to review the latest available literature concerning biomaterials, cells, and techniques used for the production of living bone grafts under in vitro conditions. To collect the data for this review, the electronic databases PubMed and Web of Science were used. The search of the available scientific reports was mostly limited to the last three years. The following term combinations/keywords were used to collect data: mesenchymal stem cells; osteoblasts; osteogenic differentiation; three-dimensional culture; scaffold; bone grafts; bioreactor; bioprinting; bioink; pulsed electromagnetic fields; bone tissue engineering; biomaterials; perfusion bioreactors; rotating bioreactors; and spinner flask bioreactors.

## 2. Current Concept of Bone Tissue Engineering 

**Figure 1:**
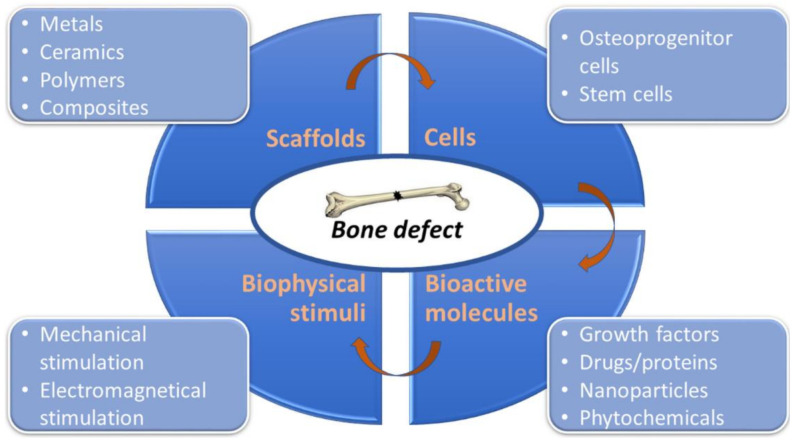
Scheme presenting the main components of bioengineered bone graft used in tissue engineering.

**Figure 2:**
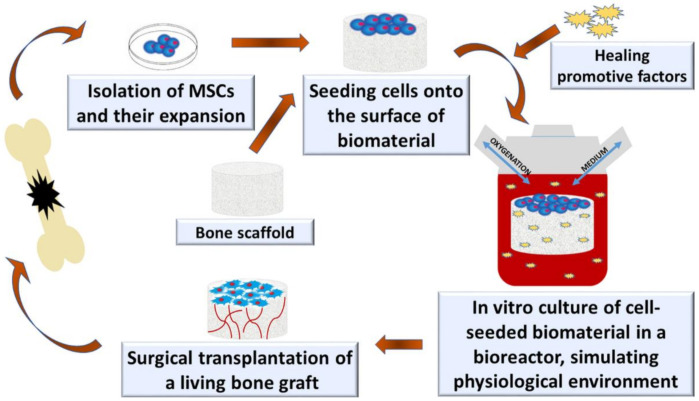
Schematic representation of the main stages of living bone graft production in vitro.

The role of BTE is an implementation of new functional bone regeneration therapy via a combination of biomaterials, cells, and healing promotive factors, such as growth factors (e.g., BMPs, PDGF, TGF-β, the insulin-like growth factor (IGF), and the fibroblast growth factor (FGF)) [18], proteins (e.g., collagen, fibronectin, and laminin) [19], drugs (e.g., antibiotics, antitumorals, or antiresorptive drugs) [20], nanoparticles (e.g., quantum dots, mesoporous silica, and gold) [21], and phytochemicals (e.g., myricetin, resveratrol, vanillic acid, and curcumin) [22]. Figure 1 shows the main components that are used in BTE for the production of bioengineered bone grafts. To successfully create living bone grafts in vitro capable of supporting bone regeneration, BTE requires the collaboration of scientists, engineers, and also orthopedic surgeons since the production of bioengineered grafts needs expertise and knowledge from different fields of science, including biology, biochemistry, materials science, and medicine [1,18,23]. The process of bone graft development and implementation may involve some or all of the following stages: (1) production of biomaterials/scaffold; (2) combination of biomaterial with healing promotive factors; (3) harvesting of bone marrow or adipose tissue from a patient and isolation of MSCs; (4) seeding cells (e.g., MSCs and osteoblasts) onto the biomaterial, followed by in vitro culture in a static condition; (5) seeding cells onto the biomaterial, followed by in vitro culture in a dynamic environment using a spinner flask (growth of premature tissue); (6) seeding cells onto the biomaterial, followed by in vitro culture in bioreactor mimicking physiological environment (growth of mature tissue); and (7) surgical transplantation of living bone graft [1,24]. Figure 2 demonstrates the main stages of bone graft development under in vitro conditions. The multidisciplinary nature of the BTE causes the translation of laboratory studies into clinical use to be generally challenging, costly, and time-consuming.

### 2.1. Biomaterials 

**Figure 3:**
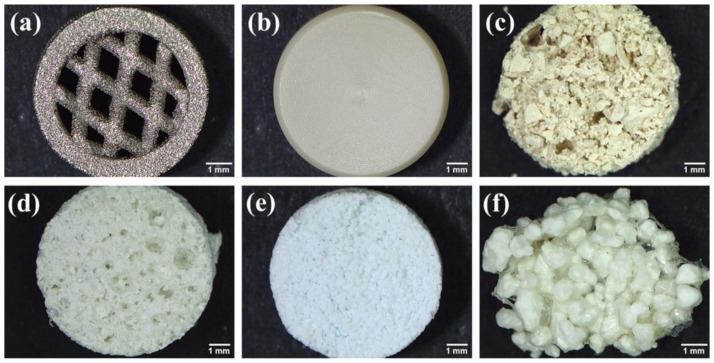
Photographs of various biomaterials that may be used for living bone graft production in vitro: (**a**) 3D printed mesh titanium alloy (Ti6Al4V); (**b**) PEEK-based biomaterial prepared by machining process; (**c**) freeze-dried chitosan/agarose/zeolite 13X composite; (**d**) freeze-dried chitosan/agarose/nanohydroxyapatite composite; (**e**) air-dried curdlan/fluoroapatite composite; and (**f**) air-dried curdlan/chitosan/hydroxyapatite composite.

Biomaterials/scaffolds for BTE applications perform a function of the platform and space for cells that will form new tissue. Microstructural properties of fabricated biomaterials should partly reflect the anatomical three-dimensional (3D) microstructure of native bone. Moreover, the biomaterials should provide temporary mechanical support at the load-bearing implantation site [13,24]. During the designing of bone scaffolds, the following criteria/features should be considered: architecture features (mechanical strength, surface topography, optimal porosity, and pore interconnectivity that ensures efficient cell colonization, oxygenation, and nutrients supply), biocompatibility (non-toxicity, non-immunogenicity, and non-genotoxicity), osteoconductivity (stimulation of cell adhesion, migration, proliferation, and bone extracellular matrix (ECM) formation), and osteoinductivity (induction of osteogenic differentiation in osteoprogenitor cells/stem cells) [25]. Ideal biomaterial for BTE applications should display a majority of the above-mentioned properties. However, it is very challenging and difficult to create ideal biomaterial. Figure 3 shows various bone scaffolds that may be used for living bone graft production in vitro.

Biomaterials used in BTE can be divided into metallic, ceramic, polymeric, and composite [13,26]. The metallic biomaterials are frequently used for dental and orthopedic applications thanks to their very good mechanical properties. The main drawback of metallic scaffolds for orthopedic applications is their poor biodegradability and high stiffness, resulting in a stress-shielding effect followed by bone atrophy and implant loosening [27]. Metallic biomaterials can be produced using stainless steel, titanium-based alloys, magnesium alloys, nickel–titanium alloys, and cobalt-based alloys [26]. In turn, ceramic materials, such as calcium phosphate cements, bioactive glass (BG), hydroxyapatite (HA), α-tricalcium phosphate (α-TCP), β-tricalcium phosphate (β-TCP), and calcium silicate, possess the ability to create direct bonds with the host bone after implantation, which is called osseointegration. Moreover, ceramic materials are characterized by good bioactivity and biodegradability. Ceramic materials may be of natural or synthetic origins [28]. Similar to ceramic materials, polymeric materials used in BTE may also occur naturally or be synthesized. Polysaccharides (chitosan, cellulose, agarose, starch, alginate, hyaluronic acid, lignin) and proteins (collagen, fibrinogen, silk, fibrin, and gelatin) are naturally derived polymers that show good biocompatibility, osteoconductivity, and low immunogenicity [13,29]. Nevertheless, they exhibit a low mechanical stability and degradation rate, which is difficult to control [13]. In turn, synthetic polymers, such as polylactic acid (PLA), polycaprolactone (PCL), poly(glycolic acid; PGA), poly(lactic acid-co-glycolic acid; PLGA), poly(ethylene glycol; PEG), polyether ether ketone (PEEK), polypropylene fumarate (PPF), polyphosphazene, and polyanhydride, are characterized by a controlled degradation rate [13]. However, unlike natural polymers, synthetic polymers exhibit a lower capability to interact with the cells [13]. 

Bone is a heterogeneous tissue consisting of mineral and organic parts. During the fabrication process of the bone scaffolds, composite biomaterials are very often created to mimic natural bone tissue, thus achieving potentially greater bioactivity [30,31]. Composite materials are composed of two or more components possessing various features to obtain biomaterials with properties that differ from particular components. The most popular composite materials are: metal–ceramic, polymer–ceramic, metal–polymer, and polymer–polymer [13,26]. Nevertheless, composites of HA and various polymers are considered as the most biomimetic materials since they were proven to significantly enhance bone formation in vitro and/or in vivo [23]. The most important features of the biomaterials used in BTE are summarized in Table 1. 

Several scaffold fabrication techniques may be distinguished in BTE, such as solvent casting/particulate leaching, porogen leaching, gas foaming, freeze-drying, melt-molding, fiber-bonding, rapid prototyping (three-dimensional (3D) printing), and electrospinning. Each production method possesses some crucial advantages, enabling the production of highly porous biomaterials that support cell distribution and growth into three-dimensional space. The selection of an appropriate technique for biomaterial synthesis depends on the expected microstructural, physicochemical, and biological properties of the final product and its planned application [32,34,35]. Recently, modern BTE most frequently uses 3D printing with living cells, which is known as the bioprinting process. Thus, the bioprinting technique will be discussed in more detail further in this review article. 

### 2.2. Cells 

In BTE, biomaterials may be used as acellular material (without cells) that supports host-cell colonization or as cellular material (seeded with the cells) that performs a role of a vehicle for cells and/or bioactive molecules. Cells may be harvested from the patient and expanded in vitro before seeding onto biomaterial [13]. The main challenge in cellular therapy is to choose an appropriate cell source that can be used to create an implant capable of repairing bone defects. There are some cell sources that may be utilized in BTE: (1) embryonic stem cells (ESCs); (2) adult stem cells, bone marrow-derived stem cells (BMDSCs), adipose tissue-derived stem cells (ADSCs), peripheral blood-derived stem cells, tooth-derived stem cells (pulp and exfoliated teeth), cord blood-derived stem cells, and amniotic fluid-derived stem cells; (3) induced pluripotent stem cells (iPSCs); and (4) bone marrow aspirate concentrate [13,23]. The choice of the type of cell depends on a facility of isolation, cell expansion efficiency, the osteogenic differentiation potential of the cells, and long-term safety, i.e., without immune rejection and carcinogenesis [23]. 

Human embryonic stem cells are pluripotent stem cells derived from the inner cell mass of the blastocyst of an embryo. These stem cells have the ability to differentiate into cells of three embryonic germ layers (ectoderm, mesoderm, and endoderm) [36]. The possibility of teratoma formation after the transplantation of embryonic stem cells into a living organism and ethical issues hinder their clinical implementation [37]. Nevertheless, pluripotency and the rapid proliferation rate of human embryonic stem cells make them appropriate for investigation purposes as a cellular model in vitro [38]. In turn, BMDSCs and ADSCs are the most common MSCs used in BTE and regenerative medicine [36]. MSCs possess the ability of multi-lineage differentiation, including the osteogenic and chondrogenic one, under standard in vitro conditions [37,39]. Moreover, it was proven that MSCs exert the immune-suppressive effect by releasing soluble factors to the microenvironment, which makes them a promising tool in accelerating bone regeneration [39]. Based on the available literature, a comparison of the osteogenic ability between BMDSCs and ADSCs showed that ADSCs have inferior osteogenic potential compared to BMDSCs. Nevertheless, due to easy accessibility in great quantities of adipose tissue as well as the good stem cells’ isolation yield and rapid proliferation rate in vitro, ADSCs may be an attractive alternative to BMDSCs for application in BTE [37]. Nevertheless, BMDSCs may be characterized by some limitations, such as immunogenic concerns (in the case of allogeneic cells), the limited availability of autologous bone marrow and their invasive harvesting procedure, and the donor age-related decrease in the cell proliferation rate [40]. Another cell source that may be utilized in BTE is the dental pulp. The dental pulp-derived MSCs are considered as a promising alternative cell source for bone regeneration since both in vitro and in vivo studies have shown that these cells reveal a high proliferation rate and a good osteogenic differentiation potential [41]. Similarly, peripheral blood-derived cells exhibited also bone regeneration efficiency [42].

To address the limitations associated with the use of MSCs, an application of iPSCs has a growing interest as a promising alternative approach to bone regeneration. The iPSCs are directly generated from somatic cells by genetic reprogramming. These cells possess the capability to differentiate into cells of three germ layers [40,43]. It was proved that iPSCs have an osteogenic differentiation ability at an equal or higher level than MSCs and unlimited self-renewal capacity. However, clinical application of iPSCs carries a risk of spontaneous teratoma formation [40]. Thus, nowadays, iPSCs-based bioengineered grafts are concerned with only laboratory-scale production and scientific purposes. 

In the last few years, bone marrow aspirate concentrate (BMAC) for regenerative medicine applications has gained significant attention due to its potential benefits in the treatment of cartilage and bone injuries. Importantly, BMAC therapy was approved by the United States Food and Drug Administration (FDA) [44]. Density gradient centrifugation of autologous bone marrow aspirate is performed to obtain BMAC, which is composed of a high concentration of MSCs, hematopoietic stem cells (HSCs), white blood cells, platelets, and growth factors, such as TGF-β, PDGF, BMP-2, and BMP-7 that are known to exert anabolic and anti-inflammatory effects [44,45]. A number of pre-clinical and clinical trials have shown the effectiveness of BMAC alone or in conjunction with platelet-rich plasma and/or biomaterials to treat musculoskeletal injuries, e.g., osteoarthritis of the knees and osteonecrosis of the femoral head. Moreover, BMAC-based bone therapy is considered as an economical method and safe due to the low risk of immune response, which makes it a promising treatment approach [45]. 

To produce functional living bone graft in vitro, it is highly recommended to seed the scaffold with various types of cells. This approach allows for creating partly vascularized bone grafts. To produce such a graft, scientists co-culture MSCs with endothelial cells (e.g., human umbilical vein endothelial cells (HUVEC) and human microvascular dermal endothelial cells (HMECs)). The endothelial cells seeded onto the 3D matrix/biomaterial reveal the ability to form vessel-like structures in vitro [46,47]. Moreover, osteo-differentiated MSCs co-cultured with endothelial cells additionally promote angiogenesis and endothelial cells’ recruitment. In turn, it was also proven that endothelial cells are able to support the osteogenic differentiation of MSCs, increasing bone formation [47]. 

### 2.3. Three-Dimensional Bioprinting 

Three-dimensional (3D) bioprinting is an additive manufacturing method used to produce biomaterials with cells or biomolecules incorporated in user-defined patterns. The desired microstructure of 3D bioprinted scaffolds is gained by the use of a computer-aided design model loaded onto a 3D printer. Next, the 3D bioprinter deposits bioink in a layer-by-layer manner to produce 3D biomaterial [36,48]. Various 3D bioprinting technologies are used to produce biomaterials, e.g., inkjet bioprinting (drop-on-demand bioprinting and electrohydrodynamic jet bioprinting) [49], micro-extrusion [50], and laser-assisted bioprinting [51]. Bioink used in tissue engineering is a material (e.g., based on natural or synthetic polymers) that possesses pre-defined rheological properties resembling the ECM. Moreover, among the desired features of bioinks, suitable viscosity range of bioinks formulation, appropriate mechanical properties, biodegradability, and high biocompatibility may be distinguished [52]. Many types of bioinks have been utilized to produce 3D biomaterials by 3D bioprinting, including polymeric materials and composite materials (polymer–polymer and polymer–ceramic). Moreover, bioinks may contain either growth factors and simulative molecules or living cells. Multicomponent bioinks have attracted wide interest due to their ability to mimic the properties of native tissue, supporting tissue regeneration after scaffold implantation [48]. It is also worth noting that the selection of the suitable cell origin for the bioprinting process guarantees successful clinical implementation of 3D bioprinted living bone grafts. Ambler et al. [53] bioprinted 3D constructs with mesenchymal progenitor cells that were isolated from different human bone sites, such as the alveolar bone, iliac crest, fibula, bone marrow, and mastoid. After 28 days of cell culture in vitro, cell viability, gene expression (ALP, COL 1, RUNX2, OCN, and OPN), and ECM mineralization were evaluated. The conducted study showed that periosteum-derived mesenchymal progenitor cells exhibited great osteogenic differentiation ability and they may be considered as a promising cell source for the production of 3D bioprinted living bone grafts in vitro.

Due to inherent versatility, printing resolution, and precision, 3D bioprinting is an attractive tool for the production of living bone grafts/substitutes for regenerative medicine. Several attempts have been made to develop bone substitutes using bioprinting. Table 2 summarizes recent studies (last three years) regarding 3D bioprinting techniques that have been used to produce living bone grafts in vitro. Chimene et al. [54] bioprinted scaffolds for craniomaxillofacial bone defects by using nanoengineered ionic covalent entanglement bioink formulation containing gelatin methacryloyl, kappa-carrageenan, nanosilicates, and human BMDSCs. They showed that the developed biomaterial stimulated the endochondral differentiation of BMDSCs and ECM mineralization. Nanosilicates are well-known as bioactive agents that have the ability to support the osteogenesis process by their products such as sodium ions, magnesium ions, lithium ions, and orthosilicic acid [55]. Liu et al. [56] fabricated functional and biomimetic nanocomposite scaffolds composed of nanosilicates, gelatin, alginate, and rat BMDSCs. They demonstrated that nanosilicates induced osteogenic differentiation of the encapsulated rat BMDSCs in vitro. Moreover, the in vivo research showed that developed 3D bioprinted biomaterial significantly supported the bone regeneration of the rat calvarial defects. In another study, Kosik-Kozioł et al. [57] showed that scaffolds composed of gelatin methacrylamide, alginate, β-TCP, and human BMDSCs increased the expression of ALP and bone gamma-carboxyglutamate protein (BGLAP) genes. In turn, Yang et al. [58] developed a novel bioink composed of collagen, human ADSCs, and neonatal chicken BMDSCs-conditioned medium that contained bioactive components supporting bone restoration, such as TGF-β and periostin. In vitro experiments showed that 3D bioprinted hADSC-based constructs increased ALP activity, ECM mineralization, and the expression of osteogenesis-related genes (runt-related transcription factor 2 (RUNX2), COL 1, ALP, BMP-2, OCN, and OPN). Developed bone constructs had also the ability to increase bone formation in vivo in a rat model. In another study, Alcala-Orozco et al. [59] produced, using bioprinting, stable hybrid constructs made of magnesium hydroxide nanoparticles-PCL and BMDSCs-laden Sr-gelatin methacrylamide. They showed that the developed novel scaffolds increased the osteogenic differentiation of encapsulated cells and ECM mineralization. Awwad et al. [60] bioprinted PLGA/PEG-based bone scaffolds comprised of the active GET (glycosaminoglycan-binding enhanced transduction system)-RUNX2 protein and human MSCs. Bioprinted scaffolds ensured controlled release of transcription factors, affecting the osteogenic differentiation of the cells. In vivo experiments conducted using a mouse model showed that implantation of 3D bioprinted biomaterial led to the development of high-density bone in a defect. Thus, they demonstrated that the incorporation of a cell-fate programming system along with viable human MSCs into a bioprinting process enabled the production of living bone graft under in vitro conditions.

Fabrication by the 3D printing technique of the template without cells, that may serve as a scaffold supporting cell adhesion, proliferation, and differentiation in vitro, is an easier approach frequently used in BTE than in the production of the scaffolds with cells already incorporated (Table 2). For example, Wang et al. [61] produced nano-attapulgite-based scaffolds by the 3D printing technique that showed induction of BMP-2 and RUNX2 gene expression in human BMDSCs. Moreover, they demonstrated, using a rat model, that fabricated novel 3D printed biomaterial enhanced bone formation. In another study, Jeong et al. [62] showed that 3D printed bone scaffolds containing gelatin and β-TCP were supportive to preosteoblasts’ (MC3T3-E1 cells) adhesion, proliferation, and differentiation in vitro. Moreover, the scaffold stimulated bone formation in the animal experiments (a rat model). Yan et al. [63] fabricated a 3D printed biodegradable PCL-based bone scaffold that had the ability to release deferoxamine (DFO). The DFO was loaded into the scaffold by immersion in the DFO solution after the bioprinting process. The DFO-loaded PCL scaffold exhibited a stimulatory effect on ECM mineralization and increased the expression of osteogenesis-related genes (RUNX2, Osterix, OCN, OPN, and COL 1) in rat BMDSCs in vitro, whereas the in vivo study using a rat model showed that a novel scaffold supported vascular ingrowth and bone regeneration.

Three-dimensional bioprinting is a rapidly emerging approach in tissue engineering and is considered as an effective and promising tool for the fabrication of living grafts. This method mainly focuses on the fabrication of cell-laden biomaterials for in vitro/in vivo studies. In situ 3D bioprinting has a growing interest in the scientific community that may revolutionize the tissue engineering field in the future. In a recent study, Li and colleagues performed the in situ restoration of a cuboid-shaped bone defect in a sacrificed rabbit by 3D scanning and 3D bioprinting using alginate hydrogel as a bioink [64]. In another study, Li et al. [65] repaired bone defects in a pig model by in situ 3D bioprinting using a robotic manipulator 3D printer and bioink containing sodium alginate, polyethylene glycol diacrylate (PEGDA), and gelatin methacrylamide. The accelerated bone repair was observed after three months. Thus, robotic-assisted 3D bioprinting in situ is a promising strategy for direct clinical applications. 

## 3. Bioreactor Systems 

Native tissue consists of micro and macroenvironments that interact with each other. A very important issue is to mimic the in vivo conditions during preclinical studies in vitro. Two-dimensional (2D) cell culture is a predominant method used in many cell-based assays. However, 2D cell culture possesses some drawbacks, e.g., poor imitation of in vivo conditions since cells are grown as a monolayer on stiff flat surfaces [34]. Recently, the 3D cell culture models have gained a growing interest in the field of tissue engineering, tumor research, and drug discovery studies. The 3D cell culture models provide appropriate cell–cell cross-talk, cell–ECM components interconnection, and intercellular signaling networks. Occurrence of the mentioned interactions within the 3D cell culture models results in better mimicry of the in vivo microenvironment compared to standard 2D cell culture [66,67]. Cells in the ideal 3D culture model should exhibit good migration, proliferation, differentiation, and cell signaling. There are three main 3D culture techniques: (1) anchorage-independent (scaffold-free), (2) anchorage-dependent (scaffold-based), and (3) specialized 3D culture platforms. The selection of an appropriate 3D cell culture model depends on the specific research direction, e.g., BTE applications and mimicry of a tumor microenvironment or a particular disease phenotype [68]. 

**Figure 4:**
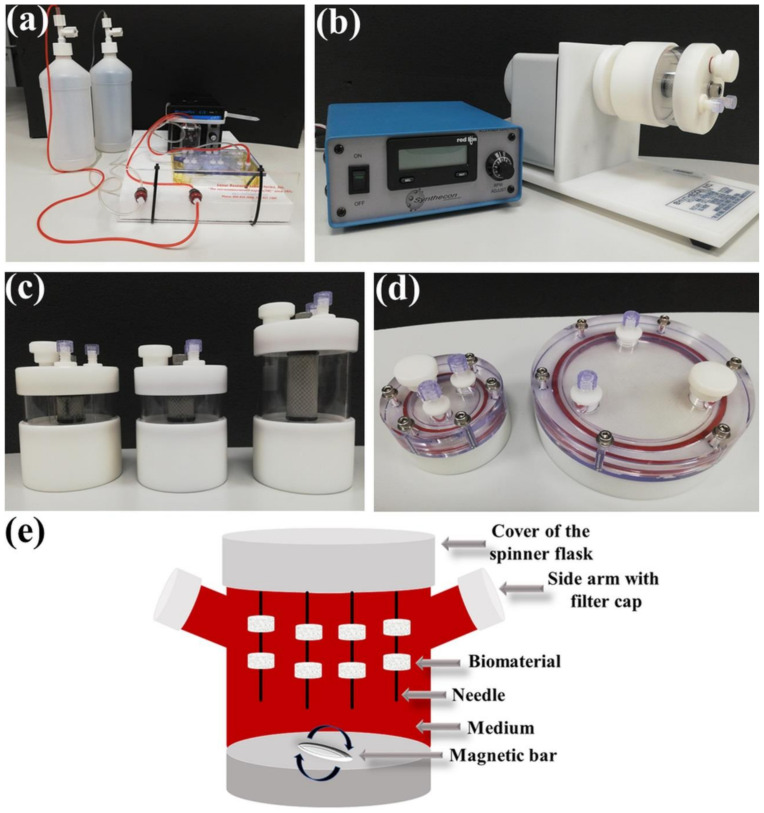
Bioreactor systems used for the production of living bone grafts in vitro: (**a**) Lazar Arrow-MTM Micro Bioreactor System (Lazar Research Laboratories, Inc., Los Angeles, CA, USA); (**b**) Rotary Cell Culture System (RCCS) (Synthecon, Houston, TX, USA) and its autoclavable vessels; (**c**) Slow Turning Lateral Vessels (STLV) and (**d**) High Aspect Ratio Vessels (HARV); and (**e**) schematic drawing of spinner flask bioreactor.

To mimic the native microenvironment of tissues under in vitro conditions, bioreactor systems are very valuable tools. Bioreactor systems can be defined as devices used to control and provide appropriate parameters during cell culture, including temperature, pH, gas and nutrient concentration, waste removal, and mechanical stimuli [69]. In bone-tissue engineering and regenerative medicine, bioreactors are excellent platforms to form living bone grafts that subsequently may develop to replace damaged bone in vivo. Additionally, bioreactors are also a good device for large-scale expansion of MSCs [70,71]. Dynamic culture conditions, obtained by using bioreactor systems, ensure good oxygenation and mass transport (of nutrients, metabolites, and waste products), as well as maintain a uniform cellular distribution and cellular survival within the graft. It is a desired phenomenon since the necrotic center within the structure of grafts cultured in the static conditions was often observed [72,73]. Moreover, dynamic culture yields shear stress, which is exerted by the medium flow, stimulating cell proliferation and differentiation [73,74]. A great diversity of dynamic 3D bioreactors has been developed for BTE applications, for example, such as perfusion bioreactors, rotating bioreactors, spinner flask bioreactors, and bioreactors with pulsed electromagnetic fields. The mentioned bioreactor systems differ in terms of cost-effectiveness, simplicity to use, monitoring options, productivity, and recommended applications [69,72,75]. Figure 4a–d demonstrate two types of commercially available bioreactor systems for BTE. The Lazar Arrow-MTM Micro Bioreactor System belongs to perfusion bioreactors with continuous medium flow whereas the Rotary Cells Culture System (RCCS) marketed by Synthecon is a rotating bioreactor. The RCCS may be used without or with biomaterials to generate 3D cell or 3D tissue models, respectively. In this section, bioreactor systems used in BTE with their general properties are outlined. Table 3 shows a summary of the studies concerning bioreactor systems used for the production of living bone grafts in vitro.

### 3.1. Perfusion Bioreactors 

Perfusion bioreactors have been developed to ensure appropriate mass transport and controlled mechanical stimuli (e.g., shear stresses and hydrodynamic forces) during 3D cell culture. The mass transfer through the interconnected pores of the 3D scaffolds and good oxygenation are obtained in the perfusion bioreactor system by the continuous flow of the cell culture medium, enhancing the cell distribution and ECM synthesis. Basic perfusion bioreactors consist of a media reservoir, a tubing circuit, a pump, perfusion/reactor chambers, a waste tank, and an oxygenator or gas-permeable membranes [69,74,94,95]. In the perfusion bioreactor, a medium is piped and next pumped to the reactor chamber containing cells/scaffold constructs. The medium may flow in a closed-loop or in an open-loop system when the bioreactor ensures a medium reservoir and a waste tank [69,96]. In these systems, the most important issue is the flow rate of the medium and fluid-induced wall shear stress that influences the microenvironment of cells and upregulates the expression of both osteoblastic markers and thus efficient bone graft formation [74,94,97]. Perfusion of the cell culture medium in bioreactors may occur indirectly or directly. Indirect perfusion occurs when the medium flows around and partly through the biomaterial, whereas direct perfusion indicates the medium flowing only through the biomaterial, thereby shear stress directly influences the cells within the scaffold [69,96,98]. The shear stress stimuli create conditions that mimic the in vivo microenvironment, enhance cell proliferation and differentiation, and also support the mineralization within the bioengineered bone tissue construct [95]. Moreover, Seddiqi et al. [99], who determined shear stress-related cell responses in vitro, showed that pulsating fluid flow with high peak shear stress (6.5 Pa) more strongly stimulated nitric oxide production by pre-osteoblasts than fluid flow with low peak shear stress (0.8 Pa). Consequently, the nitric oxide promoted the osteogenesis process by regulation of canonical Wnt/β-catenin signaling. In another study, continuous medium flow with a mean shear stress equal to 8.5 mPa improved the proliferation of human BMDSCs and increased ECM production compared to control cells cultured in static conditions [78]. In turn, Yamada et al. [79] showed that during the perfusion (shear stress distribution ranging from 0.20 mPa to 0.40 mPa), cell proliferation on polyester-based scaffolds was significantly inhibited. However, they observed increased expression of osteogenesis-related genes (RUNX2, ALP, SP7, BSP, OPN, and OCN) in the absence of chemical stimuli (i.e., the application of dexamethasone) compared to static culture. Similarly, Salifu et al. [82] proved that human fetal osteoblasts cultured on the surface of the polycaprolactone/hydroxyapatite scaffold functionalized with RGD–C (arginine–glycine–aspartate–cysteine) in a perfusion bioreactor (shear stress of 3.93 mPa) showed a lower proliferation rate but increased ALP activity and ECM mineralization.

Perfusion bioreactors are a good tool to produce bone tissue-engineered grafts for regenerative medicine applications since they provide a 3D dynamic microfluidic environment that exerts a positive effect on cellular response. For example, Panek et al. [76] cultured human BMDSCs on dexamethasone-loaded peptide hydrogels in a perfusion bioreactor. They showed that cells exhibited increased ECM mineralization and expression of osteogenesis-related genes (ALP, OCN, and COL 1). Similarly, Ressler et al. [77] showed that human BMDSCs cultured on the calcium phosphate (substituted with Mg^2+^, Zn^2+^, and SeO_3_^2−^)/chitosan composite scaffold exhibited increased COL 1 synthesis and ECM mineralization. In turn, Bhaskar et al. [81] demonstrated that human embryonic stem cell-derived mesenchymal progenitors cultured on polyurethane scaffolds in the perfusion bioreactor exhibited increased ALP activity compared to cells grown in static conditions. Gandhi et al. [80] encapsulated rat BMDSCs within fibrin beads and cultured them in the perfusion bioreactor or in static conditions for 14 days. In vitro experiments showed an increased expression of OPN, RUNX2, and VEGF in cells cultured both in static conditions and in the bioreactor. Nevertheless, in vivo studies in a rat model demonstrated that encapsulated rat BMDSCs within fibrin beads after culture in the bioreactor displayed superior mineralized bone formation and vascularization within the defect model compared with other groups. In turn, Han et al. [83] found that human fetal osteoblasts cultured on the Mg-based alloy/HA scaffold in the perfusion bioreactor synthesized higher amounts of COL 1, ALP, OCN, and OPN than cells cultured in static conditions.

Perfusion bioreactors have also been demonstrated to be a very good tool for the production of vascularized bone tissue grafts. Vascularization of the scaffold is crucial for graft survival after implantation. The promising approach in BTE is to produce pre-vascularized tissue-engineered bone grafts by using endothelial cells [75,100]. For example, Liu et al. [84] cultured human smooth muscle cells (hSMCs) on the decellularized native bone for 3 weeks and next they seeded human umbilical vein endothelial cells (HUVECs) as well as performed co-culture in a perfusion bioreactor. The developed in vitro pre-vascularization procedure allowed for obtaining a vascularized bone scaffold that was characterized by improved cellular density and better microvascular networks compared with the culture in static conditions. Moreover, it has been proved that the stromal vascular fraction (SVF) of adipose tissue may be used as a source of vasculogenic cells for the production of vascularized bone tissue grafts [101,102]. The SVF is a heterogeneous cell population that contains MSCs, preadipocytes, mature endothelial cells, and endothelial progenitor cells that promote the formation of microvascular networks [37,101]. Furthermore, to support the vascularization of the bone grafts, some investigators chose an approach to deliver pro-angiogenic factors (e.g., VEGF, PDGF, FGF, and BMP) that enhanced both new vessel and bone formation [75]. 

### 3.2. Rotating Bioreactors

In the early 1990s, the National Aeronautics and Space Administration (NASA) designed rotating bioreactor systems to simulate relative microgravity conditions. The NASA-developed Rotary Cell Culture Systems (RCCS), which are commercially accessible from Synthecon Inc. (Houston, TX, USA), have become pivotal device tools for medical studies due to their ability to provide the most favorable environment for cell and tissue cultivation [103]. It was proven that the microgravity and dynamic culture in a rotary bioreactor exert a positive effect on cell propagation, osteogenic differentiation, and mineralization [103,104,105,106,107]. The most common RCCS is composed of a rotating wall vessel, a rotary base, and a power supply (Figure 4b–d). Various designs of rotating bioreactors have been developed, e.g., cells may be seeded onto biomaterials and cultivated in a free-fall manner or biomaterials seeded with the cells are fixed on a needle in the rotating vessel during culture [69]. During constant rotation of the vessel, cells cultured on the biomaterials are maintained under a continuous circulation flow. The continuous vessel motion facilitates the exposure of the cells to gases and nutrients. Furthermore, the shear stress that is exerted by media flow may be adjusted and controlled by the rotational speed. Hence, it is possible to generate relatively low shear stress conditions [104]. 

In recent years, rotating wall vessel (RWV) bioreactors have become a popular device for tissue engineering applications. RWV bioreactors contain two concentric cylinders (inner and outer) and the cells are cultured between them. The outer cylinder rotates whereas the inner cylinder rotates or is stationary. RWV creates a microgravity environment using similar mechanisms such as RCCS bioreactors [108]. Results of the studies performed using either commercially available or custom-made rotating bioreactors are summarized in Table 3. Ravichandran et al. [85] cultured human BMDSCs on the surface of polycaprolactone/β-tricalcium phosphate-based biomaterials in a multimodal bioreactor system that allows for the application of cyclic compressive strains and biaxial rotation of a chamber. After two weeks of culture, they showed that cells exhibited increased expression of osteogenesis-related genes (ALP, OC, OCN, and COL 1) in comparison with static cultures. In turn, Koç et al. [86] applied a slow turning lateral vessel (STLV) and RCCS, along with rat BMDSCs encapsulated into microbeads, to evaluate osteogenic differentiation during dynamic culture conditions. In vitro tests proved that the encapsulated cells differentiated towards the osteoblastic lineage and formed bone-like tissue. Another study proved enhanced odontogenic differentiation of human dental pulp stem cells after dynamic culture in the RCCS bioreactor [87]. Moreover, bioreactor-incubated cell-seeded scaffolds were characterized by greater amounts of ECM proteins including glycosaminoglycans and collagen [88]. In another study, it was proven that cells cultured in a rotating/perfusion bioreactor showed a significantly higher expression of osteogenesis-related genes (RUNX2, ALP, OC, and COL 1) compared to the cells cultured in the static condition as well as in the perfusion bioreactor [89]. Thus, those observations led to a hypothesis that rotating bioreactors with continuous medium perfusion are an effective approach in BTE and may be used not only as a device for research but also as a tool for the production of living bone tissue graft.

### 3.3. Spinner Flask Bioreactors 

A spinner flask is a type of cheap and simple bioreactor that is composed of a media reservoir with two side arms with filter caps allowing for gas exchange (Figure 4e). Unlike rotating bioreactors, in a spinner flask, medium motion is created by a stirrer device. Nevertheless, similar culture conditions in the continuous circulation fluid flow are provided [69,104,109]. A magnetic bar that is placed in the middle of the flask renders the flow of fluid around the cell/biomaterial constructs. Most often, biomaterials are fixed on a needle that is linked with a cover of the spinner flask [69]. It is worth noting that the spinner flask systems provide highly homogenous medium solution on the outside of the biomaterials but the supply of oxygen and nutrients to the cells in the middle of the scaffold is less efficient compared to the perfusion bioreactors [109]. Some of the recent research that involved spinner flask bioreactors to evaluate the osteogenic differentiation of the cells cultured on the surface of bone scaffolds is presented in Table 3. Salgado et al. [91] applied a spinner flask to compare the osteogenic ability of tooth-derived stem cells for BTE applications. An increase in ALP activity and enhanced osteogenic gene expression (OC and BMP-2) compared to the static culture were observed. This effect resulted from the more efficient supply of nutrients to the cells during continuous circulation of the medium in the spinner flask. In static culture conditions, the nutrient supply was slower than in the dynamic culture. In another study, Nadine et al. [92] showed that co-encapsulated human osteoblasts and adipose-derived stromal cells in PCL-based microgels, followed by culturing in the dynamic condition in a spinner flask, may be a good method for the in vitro formation of bone-like microtissue. Moreover, microcapsules cultured in dynamic conditions exhibited enhanced ECM mineralization compared to the static cultures. In turn, Zhang et al. [93] made an attempt to fabricate functional pre-vascularized bone tissue constructs in vitro by co-culturing human amnion-derived MSCs and HUVECs on micro-carriers in the spinner flask. They observed that HUVECs exerted a negative effect on the osteogenic differentiation of MSCs, i.e., HUVECs downregulated ALP activity, ECM mineralization, and the expression of osteogenesis-related genes (COL I, RUNX2, and OC). Thus, to fabricate pre-vascularized bone microtissues, a delayed seeding method of HUVECs against MSCs should be applied. In another study, Tsai et al. [90] cultured BMDSCs on non-woven fiber disks (Fibra-Cel^®^ Disk, Eppendorf) in two different dynamic culture systems (in the spinner flask or a bidirectional-flow bioreactor). They showed that the expansion rate of cells was faster in the flow-bioreactor than in the spinner flask and static culture. Nevertheless, the highest ALP activity was observed in cells cultured in the spinner flask. Thus, spinner flask systems are a simple type of bioreactor that have exhibited good efficiency in some cases. 

### 3.4. Pulsed Electromagnetic Fields-Based Bioreactors 

Over the years, it has been observed that biophysical stimuli, such as electromagnetic stimuli, internal structural stimuli, or external mechanical stimuli, exhibit a promising potential to accelerate the regeneration of critical bone defects [110,111,112]. In the 1950s, the piezoelectric properties of the bone were discovered by a group of Japanese researchers. It was reported that bone is electropositive under tension as well as is electronegative under compression. Moreover, the formation of new bone tissue is observed in the areas of bone under tension, whereas the compression causes bone resorption. Thus, pulsed electromagnetic fields (PEMFs) have been recently implemented as an effective approach in orthopedic clinical treatment to support bone healing [113,114]. PEMFs are low-frequency magnetic fields that are generated from an alternate current. Waveform and amplitude are defined and have a permanent variation of the magnetic field amplitude over time [113]. PEMFs were approved by the FDA as a safe and non-invasive method to treat non-union or delayed-union bone fractures [113,114]. Moreover, the PEMPs are also regarded as a more efficient therapy of osteonecrosis and diabetic osteopenia in comparison with drug therapy [115]. Several studies have been performed to investigate the PEMF influence on osteoprogenitor cells in vitro and bone healing in vivo. 

It was proven that PEMFs promote MSCs’ migration and osteogenic differentiation. Additionally, PEMFs increase the secretory activity of MSCs, thereby their secretome may affect the surrounding microenvironment by the anti-inflammatory effect. Furthermore, it was shown that low-frequency (LF) PEMF downregulates the expression of proinflammatory cytokines/factors (TNF-α, IL-6, and INF-γ) and increases the level of anti-inflammatory cytokines (IL-4, IL-10, and IL-13) in vitro [112]. Activation of BMP signaling, the MAPK/ERK pathway, Notch signaling, and the Ca^2+^/CAM pathway were also observed in MSCs after PEMF stimulation. Thus, PEMFs modulate signaling pathways in MSCs that play well-established roles in bone healing [116,117,118,119]. Moreover, PEMF stimulation also significantly influences the proliferation and differentiation of osteoblastic cells. It was proven that PEMFs affect the gap junction communication system between cells and exert action on ion channels [115,120]. Importantly, in vivo studies confirmed that PEMFs significantly improve bone-implant osseointegration [110,121]. Parmaksiz et al. [122] transplanted the decellularized cancellous bone matrix (DBM) and DBM with incorporated magnetic iron oxide nanoparticles (MNPs) in the rat cranial defect model with or without LF-PEMF treatment. The in vivo study showed that implantation of DBM and DBM/MNPs in combination with LF-PEMF treatment promoted osteoblastic regeneration and angiogenesis. To translate the mentioned advantages of PEMFs to the BTE, PEMF-based bioreactors were developed. Generally, the PEMF-based bioreactor system consists of two Helmholtz coils powered by a PEMF generator, wherein a bioreactor chamber with biomaterial is placed in between them. In vitro studies indicated that cell culture in PEMF-based bioreactors enhances osteogenic differentiation and ECM mineralization [69]. For instance, Tsai et al. [123] cultured rat osteoblasts on the surface of the PLGA scaffold in a bioreactor integrated with PEMFs. The performed study showed that PEMF treatment influenced osteoblast proliferation and differentiation depending on the applied amplitudes, e.g., the 0.32 T hindered cell proliferation but increased ALP activity. 

As observed in some in vitro and in vivo studies, the PEMFs improved the osteogenic differentiation of cell/scaffold constructs compared to the static cultivation. Although PEMF-based bioreactor systems appear to be a very promising tool for the production of living bone grafts, they are characterized by high costs, limiting their wide-scale applications in BTE [69].

## 4. Conclusions

The emergence of tissue engineering and regenerative medicine at the turn of the year 1980/1990 was the hope for the revolution of treatment and therapies for patients [124]. The fast regeneration of large bone fractures is a big challenge for regenerative medicine since that type of fracture carries a risk of high mortality and morbidity. To achieve the desired healing success, the implementation of a proper bone treatment method is essential. Some commercial bone allografts and xenografts are available. Nevertheless, these bone substitutes showed some shortcomings during a precise biological evaluation, reducing their clinical relevance. Over the years, BTE has become a very promising approach, aiming to accelerate the restoration of bone defects. The main role of the BTE is the production under in vitro conditions of implantable bone substitutes via a combination of bone scaffolds/biomaterials with cells and/or bioactive molecules (e.g., growth factors). The present review described the most recent findings concerning biomaterials, cells, and techniques used for the production of living bone grafts. Viable and functional cell/scaffold constructs are crucial to achieve the desired regeneration of large bone fractures that are difficult to heal. Nevertheless, although BTE has been developing since the late 1980s and there are numerous types of biomaterials and techniques described in the literature, many proposed approaches are very complex and expensive, causing their delayed translation into clinical use. Therefore, the major challenge of BTE for the upcoming years is the development of a cost-effective and efficient method for bone tissue construct production in vitro that would allow for a significant acceleration of the bone healing process and for a rapid translation of this new technology to clinical applications. The mentioned features of living bone grafts are an important and crucial expectation for bone regenerative medicine.

It is worth noting that ideal bone scaffolds for BTE applications should reveal simultaneously good mechanical properties and high biocompatibility, which is very challenging. Moreover, ideal living bone constructs should be produced by co-culture of MSCs with endothelial cells on the surface of biomaterial in order to create pre-vascularized bone graft that will accelerate revascularization after implantation. Thus, the huge hope is that in the future, researchers will be able to design a simple and cost-effective protocol concerning the fabrication of a living bone graft under in vitro conditions. According to the available literature, 3D bioprinting is full of promise as a technique for the production of bone constructs for regenerative medicine applications due to its addressing of challenges such as high cell viability and vascularization within the bone graft. However, the use of bioprinting in clinical practice may generate high costs. In turn, bioreactor systems currently used in BTE, such as perfusion bioreactors, rotating bioreactors, and spinner flask bioreactors, are not only more cost-effective than 3D bioprinting but also they are excellent platforms to form living bone grafts under conditions mimicking the physiological microenvironment. The important advantage of bioreactor systems is the possibility to control and monitor specific parameters (biological, physical, and chemical) during the cell culture in vitro, which allows researchers to obtain replicable outcomes. However, adjustment of the respective parameters in bioreactor systems involves effort, diligence, and time. The PEMP-based bioreactors are also regarded as an efficient method for living bone graft production in vitro. Nevertheless, due to the costliness and complexity of this technology, its clinical applications are limited. Other concerns related to the clinical use of bioengineered living bone grafts are associated with law limitations, such as some strict regulations for cell-based therapies. It is worth noting that although tissue engineering and regenerative medicine have been studied for about 30 years, the production of living bone grafts is still a significant challenge for researchers.

In summary, the selection of the appropriate biomaterial and technique for the production of living bone grafts should be tailored to the needs of specific patients and type of fracture. Moreover, creation of the bioengineered bone construct is associated with cell-based therapy. Some disease entities do not allow for the collection of autologous cells from the patient. On the other hand, application of allogeneic cells may carry the risk of adverse immune response and disease transmission. Thus, production of the bone graft in vitro depends on many factors and is a very challenging task. However, taking into account the aging population and increasing demand for autologous bone transplants, it is very important to develop modern, cost-effective, simple, and universal technology for the production of bone constructs. A good compromise might be the application of cell-free bone substitutes containing bioactive molecules, promoting cell migration, proliferation, and new bone formation after transplantation in vivo. Thus, further scientific efforts should be attempted to develop efficient bone grafts that might be easily translated to clinical applications.

## Figures and Tables

**Table 1:** General features of commonly used biomaterials in BTE.

Biomaterial Type	General Features	Ref.
Metallic	Very high biomechanical load capacity and high Young’s modulus, causing stress-shielding effect, corrosion resistance, poor biodegradability, and biocompatibility	[26,32]
Ceramic	Low mechanical strength, high brittleness, slow resorption rate, biocompatibility, bioactivity, osteoconductivity, and osteoinductivity	[18,32]
Polymeric	Poor mechanical properties, low stiffness, biodegradability, biocompatibility, and low immunogenicity	[13,29,32]
Composite	Biomimetic properties, good mechanical strength, biocompatibility, osteoconductivity, osteoinductivity, bioactivity, and biodegradability	[18,31,33]

**Table 2:** Three-dimensional bioprinting techniques utilized for the production of living bone grafts in vitro.

Bioprinting Technique	Bioink	Results	Ref.
Micro-extrusion	Gelatin methacryloyl, kappa-carrageenan, nanosilicates, and human BMDSCs	In vitro (human BMDSCs): stimulated endochondral differentiation and increased ECM mineralization	[54]
Micro-extrusion	Gelatin, alginate, nanosilicates, and rat BMDSCs	In vitro (rat BMDSCs): increased ALP activity and ECM mineralization, and supported expression of osteogenesis-related genes (RUNX2, Osterix, OCN, OPN, and COL 1) In vivo (rat model): supported bone formation	[56]
Micro-extrusion	Gelatin methacrylamide, alginate, β-TCP, and human BMDSCs	In vitro (human BMDSCs): increased expression of osteogenesis-related genes (ALP and BGLAP)	[57]
Micro-extrusion	Collagen, chicken BMDSCs-conditioned medium, and human ADSCs	In vitro (human ADSCs): increased ALP activity, ECM mineralization, and expression of osteogenesis-related genes (RUNX2, COL 1, ALP, BMP-2, OCN, and OPN) In vivo (rat model): stimulated bone formation	[58]
Micro-extrusion	PCL, magnesium hydroxide nanoparticles, Sr-gelatin methacrylamide, and human BMDSCs	In vitro (human BMDSCs): increased ECM mineralization and expression of COL 1 and OCN	[59]
Micro-extrusion	PLGA, PEG, GET-RUNX, and human MSCs	In vitro (human MSCs): increased osteogenic differentiation In vivo (mouse model): supported bone formation	[60]
Micro-extrusion	Natural nano-attapulgite with polyvinyl alcohol as binder	In vitro (human BMDSCs): induced expression of osteogenesis-related genes (BMP-2 and RUNX2) In vivo (rat model): supported bone formation	[61]
Micro-extrusion	Gelatin and β-TCP	In vitro (mouse preosteoblast, MC3T3-E1 cell line): supported cell migration, proliferation, and osteogenic differentiation In vivo (rat model): stimulated bone formation	[62]
Micro-extrusion	PCL	In vitro (rat BMDSCs): increased ECM mineralization and expression of osteogenesis-related genes (RUNX2, Osterix, OCN, OPN, and COL 1) In vivo (rat model): supported vascular ingrowth and bone regeneration	[63]

**Table 3:** Bioreactor systems used for the production of living bone grafts in vitro.

Bioreactor System	Applied Physical Stimuli	Biomaterial	Cells	Results	Ref.
Perfusion	1 mL/min medium flow rate; dynamic compression (1% strain at 1 Hz)	Chitosan-graphene scaffold	Human BMDSCs	Increased cell viability and enhanced ECM mineralization	[73]
Perfusion	0.1 mL/min medium flow rate	DEX-loaded RADA 16-I scaffold	Human BMDSCs	Increased ECM mineralization and expression of osteogenesis-related genes (ALP, OCN, and COL 1)	[76]
Perfusion	1.7 mL/min medium flow rate	Calcium phosphate (substituted with Mg^2+^, Zn^2+^ and SeO_3_^2−^)/chitosan composite scaffold	Human BMDSCs	Supported COL 1 synthesis and ECM mineralization	[77]
Perfusion	1.7 mL/min medium flow rate; dynamic compression (10% strain at 1 Hz)	Human femoral head-derived decellularized bone scaffold	Human BMDSCs	Increased cell proliferation and ECM synthesis	[78]
Perfusion	1.6 mL/min medium flow rate	Poly(L-lactide-co-trimethylene carbonate) lactide (LTMC) scaffold	Rat BMDSCs	Decreased cell proliferation and increased expression of osteogenesis-related genes (RUNX2, ALP, SP7, BSP, OPN, and OCN)	[79]
Perfusion	10 mL/min medium flow rate	Fibrin beads	Rat BMDSCs	Increased expression of osteogenesis-related genes (OPN, RUNX2, and VEGF)	[80]
Perfusion	3.47 mL/min medium flow rate	Polyurethane scaffold	Human embryonic stem cell-derived mesenchymal progenitors	Increased ALP activity and cell number	[81]
Perfusion	1.6 mL/min medium flow rate; shear stress of 3.93 mPa	Polycaprolactone/hydroxyapatite (PCL/HA) scaffold functionalized with RGD–C (arginine–glycine–aspartate–cysteine)	Human fetal osteoblasts (hFOB 1.19)	Decreased cell proliferation as well as increased ALP activity and ECM mineralization	[82]
Perfusion	0.3 mL/min medium flow rate	Mg-based alloy/HA scaffold	Human fetal osteoblasts (hFOB 1.19)	Increased COL 1, ALP, OCN, and OPN synthesis	[83]
Perfusion	1 mL/min medium flow rate	Porcine decellularized native bone	Human smooth muscle cells (hSMCs) and human umbilical vein endothelial cells (HUVECs)	Improved cellular density and increased microvascular networks	[84]
Rotating	5 rpm rotation rate	Polycaprolactone–β-tricalcium phosphate (PCL-TCP) scaffold	Human BMDSCs	Increased expression of osteogenesis-related genes (ALP, OC, OCN, and COL 1)	[85]
Rotating	Not provided	Chitosan/hydroxyapatite microbeads	Rat BMDSCs	Increased OC and OPN synthesis	[86]
Rotating	Not provided	Poly(lactic-co-glycolic acid; PLGA) scaffold	Human dental pulp-derived mesenchymal stem cells	Increased COL 1 synthesis and ECM mineralization	[87]
Rotating and perfusion	1 rpm rotation rate; 1−2 mL/min medium flow rate	Gelatin-coated β-tricalcium phosphate scaffold	Buccal fat pad tissue-derived mesenchymal stem cells	Supported ECM protein synthesis	[88]
Rotating and perfusion	1 rpm rotation rate; 1−2 mL/min medium flow rate	Gelatin-coated β-tricalcium phosphate scaffold	Buccal fat pad tissue-derived mesenchymal stem cells	Increased expression of osteogenesis-related genes (RUNX2, ALP, OC, and COL 1)	[89]
Spinner flask	30 rpm stirred rate	Fibra-Cel^®^ Disk (Eppendorf)	Human BMDSCs	Increased ALP activity and decreased ECM mineralization	[90]
Spinner flask	50 rpm stirred rate	Collagen/nanohydroxyapatite/phosphoserine scaffold	Human dental pulp-derived mesenchymal stem cells and human dental follicle-derived mesenchymal stem cells	Increased ALP activity and higher osteogenic gene expression (OC and BMP-2)	[91]
Spinner flask	50 rpm stirred rate	Polycaprolactone (PCL) microparticles	Human ADSCs and human osteoblasts	Enhanced ECM mineralization	[92]
Spinner flask	50 rpm stirred rate	CultiSpher S microcarriers	Human amnion-derived MSCs and HUVECs	Downregulated ALP activity, ECM mineralization, and gene expression (COL I, RUNX2, and OC)	[93]

## Data Availability

Not applicable.

## References

[1] Velasco M.A., Narváez-Tovar C.A., Garzón-Alvarado D.A. (2015). Design, Materials, and Mechanobiology of Biodegradable Scaffolds for Bone Tissue Engineering. BioMed. Res. Int..

[2] Codrea C.I., Croitoru A.-M., Baciu C.C., Melinescu A., Ficai D., Fruth V., Ficai A. (2021). Advances in Osteoporotic Bone Tissue Engineering. J. Clin. Med..

[3] Prasadh S., Wong R.C.W. (2018). Unraveling the Mechanical Strength of Biomaterials Used as a Bone Scaffold in Oral and Maxillofacial Defects. Oral Sci. Int..

[4] Tournis S., Dede A.D. (2018). Osteogenesis Imperfecta–A Clinical Update. Metabolism.

[5] Sozen T., Ozisik L., Basaran N.C. (2017). An Overview and Management of Osteoporosis. Eur. J. Rheumatol..

[6] Tiefenbach M., Scheel M., Maier A., Gehlen M., Schwarz-Eywill M., Werner M., Siebers-Renelt U., Hammer M. (2018). Osteomalacia—Clinical Aspects, Diagnostics and Treatment. Z. Rheumatol..

[7] Maffulli N., Papalia R., Zampogna B., Torre G., Albo E., Denaro V. (2016). The Management of Osteomyelitis in the Adult. Surgeon.

[8] Xie Y., Zhang L., Xiong Q., Gao Y., Ge W., Tang P. (2019). Bench-to-Bedside Strategies for Osteoporotic Fracture: From Osteoimmunology to Mechanosensation. Bone Res..

[9] Ghiasi M.S., Chen J., Vaziri A., Rodriguez E.K., Nazarian A. (2017). Bone Fracture Healing in Mechanobiological Modeling: A Review of Principles and Methods. Bone Rep..

[10] Al-Aql Z.S., Alagl A.S., Graves D.T., Gerstenfeld L.C., Einhorn T.A. (2008). Molecular Mechanisms Controlling Bone Formation during Fracture Healing and Distraction Osteogenesis. J. Dent. Res..

[11] Oryan A., Alidadi S., Moshiri A., Maffulli N. (2014). Bone Regenerative Medicine: Classic Options, Novel Strategies, and Future Directions. J. Orthop. Surg. Res..

[12] Grassi F.R., Grassi R., Vivarelli L., Dallari D., Govoni M., Nardi G.M., Kalemaj Z., Ballini A. (2020). Design Techniques to Optimize the Scaffold Performance: Freeze-Dried Bone Custom-Made Allografts for Maxillary Alveolar Horizontal Ridge Augmentation. Materials.

[13] Roseti L., Parisi V., Petretta M., Cavallo C., Desando G., Bartolotti I., Grigolo B. (2017). Scaffolds for Bone Tissue Engineering: State of the Art and New Perspectives. Mater. Sci. Eng. C.

[14] Vangsness C.T., Wagner P.P., Moore T.M., Roberts M.R. (2006). Overview of Safety Issues Concerning the Preparation and Processing of Soft-Tissue Allografts. J. Arthrosc. Relat. Surg..

[15] Govoni M., Vivarelli L., Mazzotta A., Stagni C., Maso A., Dallari D. (2021). Commercial Bone Grafts Claimed as an Alternative to Autografts: Current Trends for Clinical Applications in Orthopaedics. Materials.

[16] Orapiriyakul W., Young P.S., Damiati L., Tsimbouri P.M. (2018). Antibacterial Surface Modification of Titanium Implants in Orthopaedics. J. Tissue Eng..

[17] Miller P.D. (2015). Management of Severe Osteoporosis. Expert Opin. Pharmacother..

[18] Perez J.R., Kouroupis D., Li D.J., Best T.M., Kaplan L., Correa D. (2018). Tissue Engineering and Cell-Based Therapies for Fractures and Bone Defects. Front. Bioeng. Biotechnol..

[19] Ashwin B., Abinaya B., Prasith T., Chandran S.V., Yadav L.R., Vairamani M., Patil S., Selvamurugan N. (2020). 3D-Poly (Lactic Acid) Scaffolds Coated with Gelatin and Mucic Acid for Bone Tissue Engineering. Int. J. Biol. Macromol..

[20] Diaz-Rodriguez P., Sánchez M., Landin M. (2018). Drug-Loaded Biomimetic Ceramics for Tissue Engineering. Pharmaceutics.

[21] Walmsley G.G., McArdle A., Tevlin R., Momeni A., Atashroo D., Hu M.S., Feroze A.H., Wong V.W., Lorenz P.H., Longaker M.T. (2015). Nanotechnology in Bone Tissue Engineering. Nanomed. Nanotechnol. Biol. Med..

[22] Valentino A., Di Cristo F., Bosetti M., Amaghnouje A., Bousta D., Conte R., Calarco A. (2021). Bioactivity and Delivery Strategies of Phytochemical Compounds in Bone Tissue Regeneration. Appl. Sci..

[23] Amini A.R., Laurencin C.T., Nukavarapu S.P. (2012). Bone Tissue Engineering: Recent Advances and Challenges. Crit. Rev. Biomed. Eng..

[24] Arvidson K., Abdallah B., Applegate L.A., Baldini N., Cenni E., Gomez-Barrena E., Granchi D., Kassem M., Konttinen Y.T., Mustafa K. (2010). Bone Regeneration and Stem Cells. J. Cell. Mol. Med..

[25] Przekora A. (2019). The Summary of the most Important Cell-Biomaterial Interactions that Need to Be Considered during in Vitro Biocompatibility Testing of Bone Scaffolds for Tissue Engineering Applications. Mater. Sci. Eng. C.

[26] Zivic F., Affatato S., Trajanovic M., Schnabelrauch M., Grujovic N., Choy K.L. (2018). Biomaterials in Clinical Practice. Advances in Clinical Research and Medical Devices.

[27] Fousová M., Vojtěch D., Kubásek J., Jablonská E., Fojt J. (2017). Promising Characteristics of Gradient Porosity Ti-6Al-4V Alloy Prepared by SLM Process. J. Mech. Behav. Biomed. Mater..

[28] Gao C., Deng Y., Feng P., Mao Z., Li P., Yang B., Deng J., Cao Y., Shuai C., Peng S. (2014). Current Progress in Bioactive Ceramic Scaffolds for Bone Repair and Regeneration. Int. J. Mol. Sci..

[29] Ioan S., Buruiana L.I., Thakur V.K., Thakur M.K., Kessler M.R. (2017). Biodegradable Polymers in Tissue Engineering. Handbook of Composites from Renewable Materials.

[30] Turnbull G., Clarke J., Picard F., Riches P., Jia L., Han F., Li B., Shu W. (2017). 3D Bioactive Composite Scaffolds for Bone Tissue Engineering. Bioact. Mater..

[31] Sachot N., Engel E., Castano O. (2014). Hybrid Organic-Inorganic Scaffolding Biomaterials for Regenerative Therapies. Curr. Org. Chem..

[32] Collins M.N., Ren G., Young K., Pina S., Reis R.L., Oliveira J.M. (2021). Scaffold Fabrication Technologies and Structure/Function Properties in Bone Tissue Engineering. Adv. Funct. Mater..

[33] Kazimierczak P., Przekora A. (2020). Osteoconductive and Osteoinductive Surface Modifications of Biomaterials for Bone Regeneration: A Concise Review. Coatings.

[34] Joseph J.S., Malindisa S.T., Ntwasa M., Mehanna R.A. (2018). Two-Dimentional (2D) and Three-Dimensional (3D) Cell Culturing in Drug Discovery. Cell Culture.

[35] Subia B., Kundu J., Kundu S.C., Eberli D. (2010). Biomaterial Scaffold Fabrication Techniques for Potential Tissue Engineering Applications. Tissue Engineering.

[36] Iaquinta M.R., Mazzoni E., Bononi I., Rotondo J.C., Mazziotta C., Montesi M., Sprio S., Tampieri A., Tognon M., Martini F. (2019). Adult Stem Cells for Bone Regeneration and Repair. Front. Cell Dev. Biol..

[37] Kazimierczak P., Syta E., Przekora A., Ginalska G. (2018). Comparison of Osteogenic Differentiation Ability between Bone Marrow-Derived Mesenchymal Stem Cells and Adipose Tissue-Derived Mesenchymal Stem Cells. Med. Ogólna Nauk. Zdrowiu.

[38] Kuhn L.T., Liu Y., Boyd N.L., Dennis J.E., Jiang X., Xin X., Charles L.F., Wang L., Aguila H.L., Rowe D.W. (2014). Developmental-Like Bone Regeneration by Human Embryonic Stem Cell-Derived Mesenchymal Cells. Tissue Eng. Part A.

[39] Alonso-Goulart V., Ferreira L.B., Duarte C.A., de Lima I.L., Ferreira E.R., de Oliveira B.C., Vargas L.N., de Moraes D.D., Silva I.B.B., Faria R.D.O. (2018). Mesenchymal Stem Cells from Human Adipose Tissue and Bone Repair: A Literature Review. Biotechnol. Res. Innov..

[40] Csobonyeiova M., Polák S., Zamborsky R., Danisovic L. (2017). iPS Cell Technologies and their Prospect for Bone Regeneration and Disease Modeling: A Mini Review. J. Adv. Res..

[41] Lee Y.-C., Chan Y.-H., Hsieh S.-C., Lew W.-Z., Feng S.-W. (2019). Comparing the Osteogenic Potentials and Bone Regeneration Capacities of Bone Marrow and Dental Pulp Mesenchymal Stem Cells in a Rabbit Calvarial Bone Defect Model. Int. J. Mol. Sci..

[42] Feng S.-W., Su Y.-H., Lin Y.-K., Wu Y.-C., Huang Y.-H., Yang F.-H., Chiang H.-J., Yen Y., Wang P.D.-Y. (2021). Small Blood Stem Cells for Enhancing Early Osseointegration Formation on Dental Implants: A Human Phase I Safety Study. Stem Cell Res. Ther..

[43] Takahashi K., Yamanaka S. (2006). Induction of Pluripotent Stem Cells from Mouse Embryonic and Adult Fibroblast Cultures by Defined Factors. Cell.

[44] Chahla J., Mannava S., Cinque M.E., Geeslin A.G., Codina D., LaPrade R.F. (2017). Bone Marrow Aspirate Concentrate Harvesting and Processing Technique. Arthrosc. Tech..

[45] Yamaguchi F.S.M., Shams S., Silva E.A., Stilhano R.S. (2019). PRP and BMAC for Musculoskeletal Conditions via Biomaterial Carriers. Int. J. Mol. Sci..

[46] Genova T., Roato I., Carossa M., Motta C., Cavagnetto D., Mussano F. (2020). Advances on Bone Substitutes through 3D Bioprinting. Int. J. Mol. Sci..

[47] Genova T., Petrillo S., Zicola E., Roato I., Ferracini R., Tolosano E., Altruda F., Carossa S., Mussano F., Munaron L. (2019). The Crosstalk Between Osteodifferentiating Stem Cells and Endothelial Cells Promotes Angiogenesis and Bone Formation. Front. Physiol..

[48] Salah M., Tayebi L., Moharamzadeh K., Naini F.B. (2020). Three-Dimensional Bio-Printing and Bone Tissue Engineering: Technical Innovations and Potential Applications in Maxillofacial Reconstructive Surgery. Maxillofac. Plast. Reconstr. Surg..

[49] Li X., Liu B., Pei B., Chen J., Zhou D., Peng J., Zhang X., Jia W., Xu T. (2020). Inkjet Bioprinting of Biomaterials. Chem. Rev..

[50] Udofia E.N., Zhou W. Microextrusion Based 3D Printing-A Review. Proceedings of the 29th Annual. International. Solid Freedom. Fabrication Symposium-An Additive Manufaxturing. Conference.

[51] Guillotin B., Ali M., Ducom A., Catros S., Keriquel V., Souquet A., Remy M., Fricain J.-C., Guillemot F. (2013). Laser-Assisted Bioprinting for Tissue Engineering. Biofabrication.

[52] Li N., Guo R., Zhang Z.J. (2021). Bioink Formulations for Bone Tissue Regeneration. Front. Bioeng. Biotechnol..

[53] Amler A.-K., Dinkelborg P., Schlauch D., Spinnen J., Stich S., Lauster R., Sittinger M., Nahles S., Heiland M., Kloke L. (2021). Comparison of the Translational Potential of Human Mesenchymal Progenitor Cells from Different Bone Entities for Autologous 3D Bioprinted Bone Grafts. Int. J. Mol. Sci..

[54] Chimene D., Miller L., Cross L.M., Jaiswal M.K., Singh I., Gaharwar A.K. (2020). Nanoengineered Osteoinductive Bioink for 3D Bioprinting Bone Tissue. ACS Appl. Mater. Interfaces.

[55] Rezwan K., Chen Q.Z., Blaker J.J., Boccaccini A.R. (2006). Biodegradable and Bioactive Porous Polymer/Inorganic Composite Scaffolds for Bone Tissue Engineering. Biomaterials.

[56] Liu B., Li J., Lei X., Cheng P., Song Y., Gao Y., Hu J., Wang C., Zhang S., Li D. (2020). 3D-Bioprinted Functional and Biomimetic Hydrogel Scaffolds Incorporated with Nanosilicates to Promote Bone Healing in Rat Calvarial Defect Model. Mater. Sci. Eng. C.

[57] Kosik-Kozioł A., Costantini M., Mróz A., Idaszek J., Heljak M., Jaroszewicz J., Kijeńska E., Szöke K., Frerker N., Barbetta A. (2019). 3D Bioprinted Hydrogel Model Incorporating β-Tricalcium Phosphate for Calcified Cartilage Tissue Engineering. Biofabrication.

[58] Yang W.S., Kim W.J., Ahn J.Y., Lee J., Ko D.W., Park S., Kim J.Y., Jang C.H., Lim J.M., Kim G.H. (2020). New Bioink Derived from Neonatal Chicken Bone Marrow Cells and Its 3D-Bioprinted Niche for Osteogenic Stimulators. ACS Appl. Mater. Interfaces.

[59] Alcala-Orozco C.R., Mutreja I., Cui X., Hooper G.J., Lim K.S., Woodfield T.B. (2021). Hybrid Biofabrication of 3D Osteoconductive Constructs Comprising Mg-Based Nanocomposites and Cell-Laden Bioinks for Bone Repair. Bone.

[60] Abu Awwad H.A.-D.M., Thiagarajan L., Kanczler J., Amer M., Bruce G., Lanham S., Rumney R., Oreffo R., Dixon J.E. (2020). Genetically-Programmed, Mesenchymal Stromal Cell-Laden & Mechanically Strong 3D Bioprinted Scaffolds for Bone Repair. J. Control. Release.

[61] Wang Z., Hui A., Zhao H., Ye X., Zhang C., Wang A., Zhang C. (2020). A Novel 3D-Bioprinted Porous Nano Attapulgite Scaffolds with Good Performance for Bone Regeneration. Int. J. Nanomed..

[62] Jeong J.E., Park S.Y., Shin J.Y., Seok J.M., Byun J.H., Oh S.H., Kim W.D., Lee J.H., Park W.H., Park S.A. (2020). 3D Printing of Bone-Mimetic Scaffold Composed of Gelatin/β-Tri-Calcium Phosphate for Bone Tissue Engineering. Macromol. Biosci..

[63] Yan Y., Chen H., Zhang H., Guo C., Yang K., Chen K., Cheng R., Qian N., Sandler N., Zhang Y.S. (2018). Vascularized 3D Printed Scaffolds for Promoting Bone Regeneration. Biomaterials.

[64] Li L., Yu F., Shi J., Shen S., Teng H., Yang J., Wang X., Jiang Q. (2017). In Situ Repair of Bone and Cartilage Defects Using 3D Scanning and 3D Printing. Sci. Rep..

[65] Li L., Shi J., Ma K., Jin J., Wang P., Liang H., Cao Y., Wang X., Jiang Q. (2020). Robotic in Situ 3D Bio-Printing Technology for Repairing Large Segmental Bone Defects. J. Adv. Res..

[66] Lv D., Hu Z., Lu L., Lu H., Xu X. (2017). Three-Dimensional Cell Culture: A Powerful Tool in Tumor Research and Drug Discovery (Review). Oncol. Lett..

[67] Justice B.A., Badr N.A., Felder R.A. (2009). 3D Cell Culture Opens New Dimensions in Cell-Based Assays. Drug Discov. Today.

[68] Langhans S.A. (2018). Three-Dimensional in Vitro Cell Culture Models in Drug Discovery and Drug Repositioning. Front. Pharmacol..

[69] Rauh J., Milan F., Günther K.-P., Stiehler M. (2011). Bioreactor Systems for Bone Tissue Engineering. Tissue Eng. Part B Rev..

[70] Bin Hassan M.N.F., Yazid M.D., Yunus M.H.M., Chowdhury S.R., Lokanathan Y., Idrus R.B.H., Ng A.M.H., Law J.X. (2020). Large-Scale Expansion of Human Mesenchymal Stem Cells. Stem Cells Int..

[71] Egger D., Tripisciano C., Weber V., Dominici M., Kasper C. (2018). Dynamic Cultivation of Mesenchymal Stem Cell Aggregates. Bioengineering.

[72] Ravichandran A., Liu Y., Teoh S.-H. (2017). Review: Bioreactor Design towards Generation of Relevant Engineered Tissues: Focus on Clinical Translation. J. Tissue Eng. Regen. Med..

[73] Lovecchio J., Gargiulo P., Vargas Luna J.L., Giordano E., Sigurjónsson Ó.E. (2019). A Standalone Bioreactor System to Deliver Compressive Load under Perfusion Flow to hBMSC-Seeded 3D Chitosan-Graphene Templates. Sci. Rep..

[74] Gardel L.S., Serra L.A., Reis R.L., Gomes M. (2014). Use of Perfusion Bioreactors and Large Animal Models for Long Bone Tissue Engineering. Tissue Eng. Part B Rev..

[75] Ng J., Spiller K., Bernhard J., Vunjak-Novakovic G. (2017). Biomimetic Approaches for Bone Tissue Engineering. Tissue Eng. Part B Rev..

[76] Panek M., Antunović M., Pribolšan L., Ivković A., Gotić M., Vukasović A., Mihalić K.C., Pušić M., Jurkin T., Marijanović I. (2019). Bone Tissue Engineering in a Perfusion Bioreactor Using Dexamethasone-Loaded Peptide Hydrogel. Materials.

[77] Ressler A., Antunović M., Teruel-Biosca L., Ferrer G.G., Babić S., Urlić I., Ivanković M., Ivanković H. (2021). Osteogenic Differentiation of Human Mesenchymal Stem Cells on Substituted Calcium Phosphate/Chitosan Composite Scaffold. Carbohydr. Polym..

[78] Pereira A., Lipphaus A., Ergin M., Salehi S., Gehweiler D., Rudert M., Hansmann J., Herrmann M. (2021). Modeling of the Human Bone Environment: Mechanical Stimuli Guide Mesenchymal Stem Cell–Extracellular Matrix Interactions. Materials.

[79] Yamada S., Yassin M.A., Schwarz T., Hansmann J., Mustafa K. (2021). Induction of Osteogenic Differentiation of Bone Marrow Stromal Cells on 3D Polyester-Based Scaffolds Solely by Subphysiological Fluidic Stimulation in a Laminar Flow Bioreactor. J. Tissue Eng..

[80] Gandhi J.K., Kao S.-W., Roux B.M., Rodriguez R.A., Tang S.-J., Fisher J.P., Cheng M.-H., Brey E.M. (2019). Perfusion Bioreactor Culture of Bone Marrow Stromal Cells Enhances Cranial Defect Regeneration. Plast. Reconstr. Surg..

[81] Bhaskar B., Owen R., Bahmaee H., Rao P.S., Reilly G.C. (2017). Design and Assessment of a Dynamic Perfusion Bioreactor for Large Bone Tissue Engineering Scaffolds. Appl. Biochem. Biotechnol..

[82] Salifu A.A., Obayemi J.D., Uzonwanne V.O., Soboyejo W.O. (2020). Mechanical Stimulation Improves Osteogenesis and the Mechanical Properties of Osteoblast-Laden RGD-Functionalized Polycaprolactone/Hydroxyapatite Scaffolds. J. Biomed. Mater. Res. Part A.

[83] Han H., Jun I., Seok H., Lee K., Lee K., Witte F., Mantovani D., Kim Y., Glyn-Jones S., Edwards J.R. (2020). Biodegradable Magnesium Alloys Promote Angio-Osteogenesis to Enhance Bone Repair. Adv. Sci..

[84] Liu X., Jakus A.E., Kural M., Qian H., Engler A., Ghaedi M., Shah R., Steinbacher D.M., Niklason L.E. (2018). Vascularization of Natural and Synthetic Bone Scaffolds. Cell Transplant..

[85] Ravichandran A., Wen F., Lim J., Chong M.S.K., Chan J.K., Teoh S. (2018). Biomimetic Fetal Rotation Bioreactor for Engineering Bone Tissues—Effect of Cyclic Strains on Upregulation of Osteogenic Gene Expression. J. Tissue Eng. Regen. Med..

[86] Demir A.K., Elçin A.E., Elçin Y.M. (2018). Osteogenic Differentiation of Encapsulated Rat Mesenchymal Stem Cells Inside a Rotating Microgravity Bioreactor: In Vitro and in Vivo Evaluation. Cytotechnology.

[87] Li Y., He L., Pan S., Zhang L., Zhang W., Yi H., Niu Y. (2016). Three-Dimensional Simulated Microgravity Culture Improves the Proliferation and Odontogenic Differentiation of Dental Pulp Stem Cell in PLGA Scaffolds Implanted in Mice. Mol. Med. Rep..

[88] Nokhbatolfoghahaei H., Paknejad Z., Bohlouli M., Rad M.R., Aminishakib P., Derakhshan S., Amirabad L.M., Nadjmi N., Khojasteh A. (2020). Fabrication of Decellularized Engineered Extracellular Matrix through Bioreactor-Based Environment for Bone Tissue Engineering. ACS Omega.

[89] Nokhbatolfoghahaei H., Bohlouli M., Paknejad Z., Rad M.R., Amirabad L.M., Salehi-Nik N., Khani M.M., Shahriari S., Nadjmi N., Ebrahimpour A. (2020). Bioreactor Cultivation Condition for Engineered Bone Tissue: Effect of Various Bioreactor Designs on Extra Cellular Matrix Synthesis. J. Biomed. Mater. Res.-Part A.

[90] Tsai H.-H., Yang K.-C., Wu M.-H., Chen J.-C., Tseng C.-L. (2019). The Effects of Different Dynamic Culture Systems on Cell Proliferation and Osteogenic Differentiation in Human Mesenchymal Stem Cells. Int. J. Mol. Sci..

[91] Salgado C.L., Barrias C.C., Monteiro F.J.M. (2020). Clarifying the Tooth-Derived Stem Cells Behavior in a 3D Biomimetic Scaffold for Bone Tissue Engineering Applications. Front. Bioeng. Biotechnol..

[92] Nadine S., Patrício S.G., Correia C.R., Mano J.F. (2019). Dynamic Microfactories Co-Encapsulating Osteoblastic and Adipose-Derived Stromal Cells for the Biofabrication of Bone Units. Biofabrication.

[93] Zhang S., Zhou M., Ye Z., Zhou Y., Tan W.-S. (2017). Fabrication of Viable and Functional Pre-Vascularized Modular Bone Tissues by Coculturing MSCs and HUVECs on Microcarriers in Spinner Flasks. Biotechnol. J..

[94] Gaspar D., Gomide V., Monteiro F. (2012). The Role of Perfusion Bioreactors in Bone Tissue Engineering. Biomatter.

[95] Birru B., Mekala N.K., Parcha S.R. (2019). Mechanistic role of perfusion culture on bone regeneration. J. Biosci..

[96] Ceccarelli G., Bloise N., Vercellino M., Battaglia R., Morgante L., Gabriella Cusella De Angelis M., Imbriani M., Visai L. (2013). In Vitro Osteogenesis of Human Stem Cells by Using a Three-Dimensional Perfusion Bioreactor Culture System: A Review. Recent Pat. Drug Deliv. Formul..

[97] Zhao F., Van Rietbergen B., Ito K., Hofmann S. (2020). Fluid Flow-Induced Cell Stimulation in Bone Tissue Engineering Changes due to Interstitial Tissue Formation in Vitro. Int. J. Numer. Methods Biomed. Eng..

[98] Bancroft G.N., Sikavitsas V.I., Mikos A.G. (2003). Technical Note: Design of a Flow Perfusion Bioreactor System for Bone Tissue-Engineering Applications. Tissue Eng..

[99] Seddiqi H., Saatchi A., Amoabediny G., Helder M.N., Ravasjani S.A., Aghaei M.S.H., Jin J., Zandieh-Doulabi B., Klein-Nulend J. (2020). Inlet Flow Rate of Perfusion Bioreactors Affects Fluid Flow Dynamics, but Not Oxygen Concentration in 3D-Printed Scaffolds for Bone Tissue Engineering: Computational Analysis and Experimental Validation. Comput. Biol. Med..

[100] O’Brien F.J. (2011). Biomaterials & Scaffolds for Tissue Engineering. Mater. Today.

[101] Born G., Nikolova M., Scherberich A., Treutlein B., García-García A., Martin I. (2021). Engineering of Fully Humanized and Vascularized 3D Bone Marrow Niches Sustaining Undifferentiated Human Cord Blood Hematopoietic Stem and Progenitor Cells. J. Tissue Eng..

[102] Ismail T., Lunger A., Haumer A., Todorov A., Menzi N., Schweizer T., Bieback K., Bürgin J., Schaefer D.J., Martin I. (2020). Platelet-Rich Plasma and Stromal Vascular Fraction Cells for the Engineering of Axially Vascularized Osteogenic Grafts. J. Tissue Eng. Regen. Med..

[103] Mazzoleni G., Boukhechba F., Steimberg N., Boniotti J., Bouler J.M., Rochet N. (2011). Impact of Dynamic Culture in the RCCSTM Bioreactor on a Three-Dimensional Model of Bone Matrix Formation. Procedia Eng..

[104] Hansmann J., Groeber F., Kahlig A., Kleinhans C., Walles H. (2012). Bioreactors in Tissue Engineering-Principles, Applications and Commercial Constraints. Biotechnol. J..

[105] Facer S.R., Zaharias R.S., Andracki M.E., Lafoon J., Hunter S., Schneider G.B. (2005). Rotary Culture Enhances Pre-Osteoblast Aggregation and Mineralization. J. Dent. Res..

[106] Penolazzi L., Lolli A., Sardelli L., Angelozzi M., Lambertini E., Trombelli L., Ciarpella F., Vecchiatini R., Piva R. (2016). Establishment of a 3D-Dynamic Osteoblasts–Osteoclasts Co-Culture Model to Simulate the Jawbone Microenvironment in Vitro. Life Sci..

[107] Grimm D., Egli M., Krüger M., Riwaldt S., Corydon T.J., Kopp S., Wehland M., Wise P., Infanger M., Mann V. (2018). Tissue Engineering Under Microgravity Conditions–Use of Stem Cells and Specialized Cells. Stem Cells Dev..

[108] Swaminathan V., Bechtel G., Tchantchaleishvili V. (2021). Artificial Tissue Creation under Microgravity Conditions: Considerations and Future Applications. Artif. Organs.

[109] Yeatts A., Fisher J.P. (2011). Bone Tissue Engineering Bioreactors: Dynamic Culture and the Influence of Shear Stress. Bone.

[110] Hao Z., Xu Z., Wang X., Wang Y., Li H., Chen T., Hu Y., Chen R., Huang K., Chen C. (2021). Biophysical Stimuli as the Fourth Pillar of Bone Tissue Engineering. Front. Cell Dev. Biol..

[111] Hatefi S., Alizargar J., Le Roux F., Hatefi K., Sh M.E., Davids H., Hsieh N.-C., Smith F., Abou-El-Hossein K. (2021). Review of Physical Stimulation Techniques for Assisting Distraction Osteogenesis in Maxillofacial Reconstruction Applications. Med. Eng. Phys..

[112] Ross C.L., Syed I., Smith T.L., Harrison B.S. (2017). The Regenerative Effects of Electromagnetic Field on Spinal Cord Injury. Electromagn. Biol. Med..

[113] Caliogna L., Medetti M., Bina V., Brancato A., Castelli A., Jannelli E., Ivone A., Gastaldi G., Annunziata S., Mosconi M. (2021). Pulsed Electromagnetic Fields in Bone Healing: Molecular Pathways and Clinical Applications. Int. J. Mol. Sci..

[114] Yuan J., Xin F., Jiang W. (2018). Underlying Signaling Pathways and Therapeutic Applications of Pulsed Electromagnetic Fields in Bone Repair. Cell. Physiol. Biochem..

[115] Wang T., Yang L., Jiang J., Liu Y., Fan Z., Zhong C., He C. (2019). Pulsed Electromagnetic Fields: Promising Treatment for Osteoporosis. Osteoporos. Int..

[116] Varani K., Vincenzi F., Pasquini S., Blo I., Salati S., Cadossi M., De Mattei M. (2021). Pulsed Electromagnetic Field Stimulation in Osteogenesis and Chondrogenesis: Signaling Pathways and Therapeutic Implications. Int. J. Mol. Sci..

[117] Leone L., Podda M.V., Grassi C. (2015). Impact of Electromagnetic Fields on Stem Cells: Common Mechanisms at the Crossroad between Adult Neurogenesis and Osteogenesis. Front. Cell. Neurosci..

[118] Ross C.L., Siriwardane M., Almeida-Porada G., Porada C.D., Brink P., Christ G.J., Harrison B.S. (2015). The Effect of Low-Frequency Electromagnetic Field on Human Bone Marrow Stem/Progenitor Cell Differentiation. Stem Cell Res..

[119] Ross C.L., Ang D.C., Almeida-Porada G. (2019). Targeting Mesenchymal Stromal Cells/Pericytes (MSCs) With Pulsed Electromagnetic Field (PEMF) Has the Potential to Treat Rheumatoid Arthritis. Front. Immunol..

[120] Galli C., Pedrazzi G., Guizzardi S. (2019). The Cellular Effects of Pulsed Electromagnetic Fields on Osteoblasts: A Review. Bioelectromagnetics.

[121] Fini M., Giavaresi G., Setti S., Martini L., Torricelli P., Giardino R. (2004). Current Trends in the Enhancement of Biomaterial Osteointegration: Biophysical Stimulation. Int. J. Artif. Organs.

[122] Parmaksiz M., Lalegül-Ülker Ö., Vurat M.T., Elçin A.E., Elçin Y.M. (2021). Magneto-Sensitive Decellularized Bone Matrix with or without Low Frequency-Pulsed Electromagnetic Field Exposure for the Healing of a Critical-Size Bone Defect. Mater. Sci. Eng. C.

[123] Tsai M.-T., Chang W.H.-S., Chang K., Hou R.-J., Wu T.-W. (2007). Pulsed Electromagnetic Fields Affect Osteoblast Proliferation and Differentiation in Bone Tissue Engineering. Bioelectromagnetics.

[124] Nerem R.M. (2006). Tissue Engineering: The Hope, the Hype, and the Future. Tissue Eng..

